# New Remote Cerebral Microbleeds on T2^*^-Weighted Echo Planar MRI After Intravenous Thrombolysis for Acute Ischemic Stroke

**DOI:** 10.3389/fneur.2021.744701

**Published:** 2022-02-15

**Authors:** Bartosz Jabłoński, Anna Gójska-Grymajło, Daria Ossowska, Edyta Szurowska, Adam Wyszomirski, Bartłomiej Rojek, Bartosz Karaszewski

**Affiliations:** ^1^Department of Adult Neurology, University Clinical Center, Medical University of Gdańsk, Gdańsk, Poland; ^2^II Department of Radiology, Medical University of Gdańsk, Department of Radiology, University Clinical Centre, Gdańsk, Poland

**Keywords:** cerebral microbleeds, acute ischemic stroke, thrombolysis, hemorrhagic transformation, neuroimaging, MRI

## Abstract

**Background:**

The main and well-defined complication of intravenous administration of recombinant tissue plasminogen activator (tPA) in patients with acute ischemic stroke (AIS) is symptomatic intracranial hemorrhage (sICH). However, rtPA might also be connected with the formation of cerebral microbleeds (CMBs), located remotely from the ischemic lesions, that may remain clinically silent. This association might be important because the load of CMBs has been associated with cognitive impairment. We investigated whether administration of rtPA in AIS results in the appearance of new CMBs and if the initial load of CMBs is associated with hemorrhagic transformation.

**Methods:**

A total of fifty-nine consecutive patients with AIS treated with rtPA underwent MRI including T2^*^-weighted Echo Planar Imaging (T2^*^-EPI) shortly before and 7–9 days after rtPA administration. We calculated the load of new CMBs located outside the MR diffusion restriction area in the follow-up imaging and assessed hemorrhagic transformation with ECASS-II scoring.

**Results:**

A total of forty-nine patients were included for the final analysis. On initial T2^*^-EPI-GRE, 37 baseline microbleeds (CMBs) were observed in 14 patients (28.6%). On follow-up T2^*^-EPI-GRE amount of CMBs increased to a total number of 103. New CMBs were found in 5 (14.3%) of 35 patients without and in 9 (64.3%) of 14 with any baseline CMBs. Multiple logistic regression analysis indicated that presence of baseline CMBs (risk ratio [RR] 5.95, 95% CI 2.69–13.20, *p* < 0.001) and lower platelets level (risk ratio [RR] 0.992, 95% CI 0.986–0.998, *p* = 0.007) were independently associated with new CMBs. The baseline load of CMBs was not associated with the risk of hemorrhagic transformation.

**Conclusion:**

In this study, new CMBs were found in nearly 30% of patients with AIS on the 7–9 days after rtPA treatment. Baseline CMBs correlated with a higher risk of new CMBs appearing after the rtPA treatment, independently of other factors. At the same time, in our sample, baseline CMBs did not correlate with an increased risk of hemorrhagic transformation. Since the associations between the CMBs load and cognitive impairment have already been proved, further studies are warranted to investigate possible associations between the thrombolytic treatment of patients with AIS, mainly those with baseline CMBs, and the risk of earlier cognitive decline.

## Introduction

Intravenous thrombolysis with recombinant tissue plasminogen activator (tPA) is the mainstay therapeutic method of acute ischemic stroke (AIS) with proven clinical benefit (1) and is recommended up to 4.5 h after stroke onset (2). In addition, recent randomized controlled trials and meta-analyses enabled the extension of the time window in selected cases or administration of rtPA in some subjects with unknown onset of stroke (3–5).

The most common complication of rtPA treatment is the hemorrhagic transformation of the ischemic lesion. This transformation might be of various severity that has been classified with ECASS-II score (Table 1) (6). The severity of hemorrhagic transformation is strictly connected with clinical significance and the symptomatic intracerebral hemorrhage (sICH), which occurs in 3–7% of rtPA treated patients, is of the highest clinical interest since it is associated with poor functional outcomes. Symptomatic intracerebral hemorrhage has been well-characterized in the multiple studies (2, 7–9).

**Table 1:** The european cooperative acute stroke study classification of hemorrhagic transformation.

**Hemorrhage classification**	**Radiographic appearance**
Haemorrhagic infarction type 1 (HI1)	Small hyperdense petechiae.
Haemorrhagic infarction type 2 (HI2)	More confluent hyperdensity throughout the infarct zone; without mass effect.
Parenchymal hematoma type 1 (PH1)	Homogeneous hyperdensity occupying <30% of the infarct zone; some mass effect.
Parenchymal hematoma type 2 (PH2)	Homogeneous hyperdensity occupying >30% of the infarct zone; significant mass effect. Or, any homogenous hyperdensity located beyond the borders of the infarct zone.

Administration of rtPA might also result in another hemorrhagic complication—cerebral microhemorrhages or microbleeds (CMBs). CMBs are small, rounded signal loss lesions surrounded by brain tissue with a diameter up to 5 mm. CMBs might appear remotely from the ischemic lesion in an isolated or diffuse pattern across the brain. The CMBs neuroimaging characteristics are listed in Table 2 (10–13). The prevalence of CMBs is significantly higher in the elderly population with multiple comorbidities and high-total cardiovascular risk, especially hypertension with subsequent hypertensive arteriopathy (13, 14). Usually, CMBs are clinically silent in the terms of acute stroke care but might have a cumulative impact on patients in the following years, mainly because of the associations between the load of CMBs and cognitive impairment (15–20).

**Table 2:** Cerebral microbleeds (CMBs)—neuroimaging characteristics.

**Small, rounded signal loss lesions surrounded by brain tissue**
Located outside the infarcted area
Diameter up to 5 mm
Detected on T2*-weighted and susceptibility-weighted imaging (SWI)
Blooming effect on T2*-weighted MRI
Generally not seen on computed tomography, FLAIR, T1-weighted MRI

Cerebral microbleeds have also been under study because of their possible pathophysiological connection with hemorrhagic transformation as CMBs are commonly treated as markers of increased vascular vulnerability due to severe small vessel disease (21–23). This connection seems plausible also in light of the recent analyses that have revealed that CMBs burden might be a useful single marker of the risk of sICH in patients, after stroke or TIA, receiving oral anticoagulants (OACs) (24, 25). In these reports, CMBs burden was more predictive of sICH than other tools, including HAS-BLED (26).

The problem of rtPA-associated CMBs has already been addressed in a few studies (27–32). However, only four of them were planned to assess true baseline CMBs load with MRI preceding the rtPA administration (28–31). The problem to quantify and compare CMBs before and after the thrombolytic treatment is related to the fact that their proper assessment requires the use of MRI. The long duration of standard MRI testing interferes with the urge of administering the rtPA and thus precludes regular and easy use of this neuroimaging technique in thrombolysed patients.

In the presented study, we investigated whether administration of rtPA in AIS results in the appearance of new CMBs and whether the baseline CMBs load increases the risk of new CMBs and hemorrhagic transformation after the rtPA treatment. The assessment was performed using pre- and post-thrombolysis special MRI protocols.

## Methods

### Patients and Study Protocol

A total of fifty-nine consecutive patients with AIS treated with intravenous thrombolysis between March 20, 2019 and October 19, 2020 were prospectively enrolled into the study. The study protocol included the following:

(1) head MRI that includes T2^*^-weighted Echo Planar Imaging (T2^*^-EPI-GRE) in the protocol before rtPA administration (to exclude ICH);
(2) intravenous thrombolysis, as the main therapy (without mechanical thrombectomy) with standard dosage of rtPA administered either within 4.5 h after stroke onset (the time when a patient was last known to be without symptoms), according to standard dosing protocol approved and recommended for thrombolysis in patients with AIS (33) or with unknown time of onset based on WAKE-UP trial protocol (3) with DWI-FLAIR MRI mismatch (five patients).
(3) Follow-up MRI that includes T2^*^-EPI-GRE in the protocol, performed on 7–9 days after stroke onset (for therapeutic reasons, in eight cases, follow-up MRI was performed out of the target time frame).

The aforementioned steps constituted the eligibility criteria—patients who could not follow one or more of the steps were not included in the assessments. In addition, the exclusion criterion was the poor quality of obtained neuroimaging data. Forty-nine patients (26 women and 23 men, mean age - 66 years) were included in the final analysis. Three of them had been treated with the non-vitamin K antagonist oral anticoagulant (NOAC) before stroke onset because of atrial fibrillation. However, their laboratory-assessed anticoagulant activity on admission was low (<20 ng/ml) and did not constitute a contraindication for thrombolysis. None of the patients involved in the analysis received any kind of anticoagulation before the follow-up MRI.

All the patients provided informed consent for the involvement in the study. The clinical characteristics of the study group are presented in Table 3.

**Table 3:** Characteristics of two groups – with (*N* = 14) and without (*N* = 35) new CMBs.

	**1 (*N* = 14)**	**0 (*N* = 35)**	**Total (*N* = 49)**	***p* value**
Age, y, median (Q1, Q3)	72.5 (66.5, 84.0)	62.0 (46.0, 75.5)	66.0 (56.0, 80.0)	**0.026**
Time from onset to treatment, min, mean (SD)	177.4 (63.3)	170.9 (59.5)	172.8 (60.0)	0.734
Hypertension	13 (92.9%)	27 (77.1%)	40 (81.6%)	0.415^fe^
Diabetes	6 (42.9%)	11 (31.4%)	17 (34.7%)	0.448
Atrial fibrillation	4 (28.6%)	4 (11.4%)	8 (16.3%)	0.202^fe^
Antiplatelet drugs	5 (35.7%)	15 (42.9%)	20 (40.8%)	0.646
NOAC on admission	2 (14.3%)	1 (2.9%)	3 (6.1%)	0.193^fe^
Previous clinical stroke	3 (21.4%)	7 (20.0%)	10 (20.4%)	0.999^fe^
Systolic blood pressure admission, mmHg, mean (SD)	164.1 (21.6)	154.5 (26.1)	157.2 (25.1)	0.226
Diastolic blood pressure admission, mmHg, mean (SD)	86.5 (15.5)	84.6 (13.7)	85.2 (14.1)	0.679
Glucose, mg/dl, median (Q1, Q3)	126.0 (104.5, 141.2)	121.0 (105.0, 142.0)	121.0 (104.0, 143.0)	0.715^m−w^
Platelets, x10^∧^9/l, median (Q1, Q3)	222.0 (182.5, 240.8)	234.0 (192.5, 296.5)	231.0 (182.0, 266.0)	0.250^m−w^
Creatinine, mg/dl, mean (SD)	1.0 (0.2)	0.9 (0.2)	0.9 (0.2)	**0.035**
INR, mean (SD)	1.0 (0.1)	1.0 (0.1)	1.0 (0.1)	0.827
NIHSS score, admission, median (Q1, Q3)	5.0 (4.0, 5.8)	5.0 (3.0, 10.0)	5.0 (3.0, 7.0)	0.600^m−w^
NIHSS score, discharge, median (Q1, Q3)	0.5 (0.0, 1.8)	1.0 (0.0, 2.0)	1.0 (0.0, 2.0)	0.698^m−w^
TOAST classification				0.624^fe^
LAA	2 (14.3%)	7 (20.0%)	9 (18.4%)	
CE	5 (35.7%)	6 (17.1%)	11 (22.4%)	
SVD	3 (21.4%)	9 (25.7%)	12 (24.5%)	
UE	4 (28.6%)	13 (37.1%)	17 (34.7%)	
Hemorrhagic transformation, ECASS				0.647^fe^
HI1	3 (21.4%)	3 (8.6%)	6 (12.2%)	
HI2	1 (7.1%)	1 (2.9%)	2 (4.1%)	
NH	10 (71.4%)	29 (82.9%)	39 (79.6%)	
PH1	0 (0.0%)	1 (2.9%)	1 (2.0%)	
PH2	0 (0.0%)	1 (2.9%)	1 (2.0%)	
Baseline DWI Volume, ml, median (Q1, Q3)	2.2 (0.0, 13.4)	3.9 (0.6, 19.5)	3.4 (0.0, 16.0)	0.584^m−w^
Periventricular and deep white matter hyperintensities [2–3 in Fazekas scale]	5 (35.7%)	8 (22.9%)	13 (26.5%)	0.357
Presence of baseline CMBs	9 (64.3%)	5 (14.3%)	14 (28.6%)	**<** **0.001**

*SD, standard deviation; Q1, the first quartile; Q3, the third quartile; fe, Fisher's exact test; m-w, Mann-Whitney test; ICH, intracerebral hemorrhage; TOAST, Trial of Org 10172 in Acute Stroke Treatment (classification); LAA, large-artery atherosclerosis; CE, cardioembolism, SVD, small-vessel disease; UE- stroke of undetermined etiology; ECASS, European Cooperative Acute Stroke Study (classification); NH, no hemorrhage; HI1, haemorrhagic infarction type 1; HI2, hemorrhagic infarction type 2; PH1, parenchymal hematoma type 1; PH2, parenchymal hematoma type 2; NOAC, novel oral anticoagulant; NIHSS, National Institute of Health Stroke Scale; SBP, systolic blood pressure; DBP, diastolic blood pressure; DWI, diffusion weighted imaging. Statistically significant differences between these two groups of patients are marked with bald fonts of the p-value*.

### MRI Protocol and Image Analysis

All MRI examinations were performed with a 1.5-T MRI scanner (Magnetom Aera, Siemens, Erlangen, Germany) with 20 channel head/neck coil in AutoCoil selection mode. Baseline MRI—the “Go-Brain” protocol—designed for the fast imaging in the acute phase of stroke (34), consisted of sagittal T1-weighted gradient recalled echo (GRE), axial T2-weighted turbo spin-echo (TSE), axial T2-weighted TSE fluid-attenuation inversion recovery (FLAIR), axial diffusion-weighted (DWI) single-shot echo-planar imaging (EPI), and axial T2^*^-weighted EPI-GRE. The high-diagnostic value of ultrafast sequences was presented by Prakkamakul et al. (35). The “Go-Brain” protocol included the “AutoAlign” mode, which uses anatomical landmarks for automated alignment for slice position and prevents erroneous double counting in the follow-up assessment.

The follow-up MRI protocol included axial T2^*^-weighted EPI-GRE sequences that were used to assess the number of CMBs. Next, where needed the susceptibility-weighted imaging (SWI) was used to confirm CMBs detected on the follow-up T2^*^-weighted EPI-GRE images. DWI sequence was also used to evaluate baseline (before rtPA administration) infarct volume. Axial T2 FLAIR sequence was used to assess the leukoaraiosis severity in the Fazekas scale (36). Detailed MRI parameters are listed in Table 4.

**Table 4:** MRI sequences and parameters used in the study.

**Sequence**	**TR [ms]**	**TE [ms]**	**IT [ms]**	**Slices**	**Slice thickness [mm]**	**Gap [mm]**	***b*-value**
Sagital T1 (GRE)	595	11	-	27	5	1	-
Axial T2 (TSE)	4,700	101	-	25	5	1	-
Axial T2 FLAIR	5,500	78	1,930	25	5	1	-
Axial T2* (EPI-GRE)	6,120	75	-	25	5	1	-
Axial DWI (EPI)	4,500	89	-	31	5	0.6	0.800
3D SWI (GRE)	49	40	-	56	2	-	-

*GRE, gradient recalled echo; TSE, turbo spin echo; FLAIR, fluid-attenuated inversion recovery; EPI, echo-planar imaging; DWI, diffusion-weighted imaging; SWI, susceptibility-weighted imaging*.

Cerebral microbleeds neuroimaging characteristics are listed in Table 2 (10–13). CMBs observed in the region of the ischemic lesion were not included in the analysis. CMBs in T2^*^-weighted EPI-GRE sequences were manually assessed by three observers—a neurologist (BJ) and a radiologist working together with a physicist (ESz, DO). Discrepancies were resolved by consensus and by independent decisions of an experienced stroke neurologist (BK).

Hemorrhagic transformation was graded by the ECASS-II (European Cooperative Acute Stroke Study) classification (6) (Table 1). Symptomatic intracerebral hemorrhage was defined according to SITS-MOST (The Safe Implementation of Thrombolysis in Stroke- Monitoring Study) criteria (37): a type 2 parenchymal hemorrhage (PH2) with deterioration in the National Institutes of Health Stroke Scale (NIHSS) score of 4 points or more, or death.

### Clinical Assessment

All the patients enrolled were clinically assessed by an experienced stroke neurologist and classified according to the Trial of Org 10172 in Acute Stroke Treatment (TOAST) classification (38).

### Statistics

Interrater agreement for the MRI reading was evaluated using the weighted Cohen's kappa with linear weights and interpreted in the following way: 0.01–0.20 as poor agreement, 0.21–0.40 as fair agreement, 0.41–0.60 as moderate agreement, 0.61–0.80 as substantial agreement, and above 0.80 as almost perfect agreement.

The characteristics of the patients were presented according to mean and standard deviation (SD) for normally distributed continuous data, whereas non-normally distributed variables were described as quartiles; categorical variables were reported as counts and percentages. The between-group differences were evaluated using *t*-test or Mann-Whitney test for continuous data and the chi-square test or Fisher's exact test for categorical variables. The two-tailed tests were carried out at a significance level of *p* ≤ 0.05. A generalized linear model assuming Poisson distribution with a log-link function was used to identify risk factors for the incidence of new CMBs. A stepwise forward selection was applied to build up a model with the lowest score of the Akaike information criterion. The final model was recalculated taking into account a robust error variance. Regression coefficients were expressed as adjusted relative risk (aRR) with a 95% CI. All the statistical analyses were performed using the R statistical package (version 3.6.3.).

## Results

**Figure 1:**
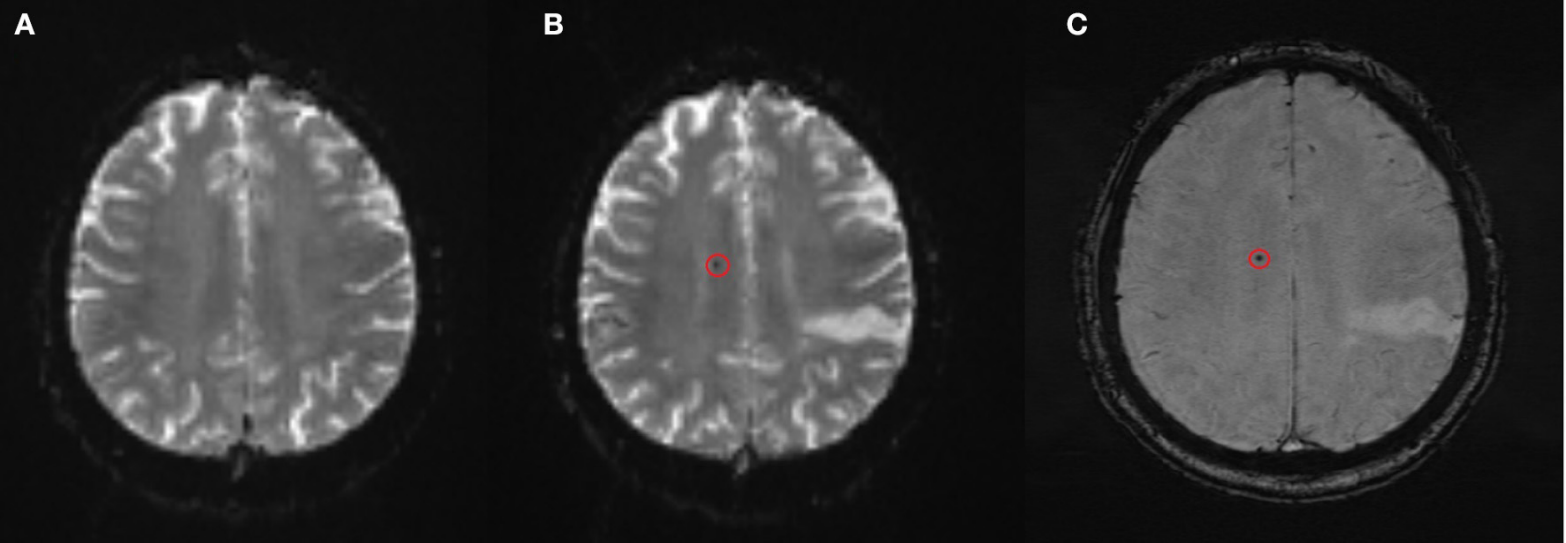
New CMB found on the follow-up MRI on day 7–9 after rtPA treatment **(A)** baseline T2*-weighted EPI-GRE sequence, **(B)** follow-up T2*-weighted EPI-GRE, **(C)** susceptibility-weighted imaging confirmatory slice.

On initial T2^*^-EPI-GRE we identified 37 baselines CMBs in 14 patients (28.6%). A total of thirty-four (91.9%) of CMBs were localized in the cortical/subcortical area and only 3 (8.1%) in the deep brain structures. In all but one patient, the number of baseline CMBs did not exceed 3. Interobserver agreement was high and kappa values are presented in Table 5. On follow-up T2^*^-EPI-GRE, the total amount of CMBs increased up to an absolute number of 103 (all patients combined). New CMBs were found in 5 (14.3%) of 35 patients without baseline CMBs (Figure 1) and in 9 (64.3%) of 14 with CMBs detected in baseline MRI. Among a total of 66 new CMBs, 48 (72.7%) were localized in the cortical/subcortical area, 18 (27.3%) in deep brain structures, and 35 (53.0%) in the ipsilateral hemisphere. Hemorrhagic transformation (ECASS) was observed in 10 (20.4%) patients - 6 HI1, 2 HI2, 1 PH1, and 1 PH2. Baseline CMBs did not correlate significantly with hemorrhagic transformation (*p* = 0.647).

**Table 5:** Inter-observer agreement for the MRI assessment of CMBs.

**CMBs on MRI**	Baseline [kappa]^*^	Follow-up [kappa]^*^
Deep structures	0.64	0.78
Cortical/subcortical	0.58	0.74
All	0.63	0.77

* * interobserver agreement was evaluated using the weighted Cohen's kappa with linear weights and interpreted in the following way: 0.01–0.20 as poor agreement, 0.21–0.40 as fair agreement, 0.41–0.60 as moderate agreement, 0.61–0.80 as substantial agreement, and above 0.80 as almost perfect agreement. The interobserver agreement was evaluated between the assessments of the neurologist (BJ) and the radiology team (ES and DO)*.

Patients with new CMBs were older (median 72.5, interquartile range (IQR) 66.5, 84.0 vs. 62.0, IQR 46.0, 75.5; *p* = 0.026), had higher creatinine level (median 1.0, IQR 0.9,1.1 vs. 0.8, IQR 0.7, 0.9; *p* = 0.035) and more often had higher counts of baseline CMBs: 9 (64.3%) vs. 5 (14.3%), *p* < 0.001. Other parameters were not statistically significantly different between the groups including pre-existing hypertension, the National Institutes of Health Stroke Scale (NIHSS) admission score, stroke subtype according to TOAST classification, the severity of leukoaraiosis, and baseline DWI lesion volume.

Multiple logistic regression analysis (Table 6) indicated that presence of baseline CMBs (risk ratio 5.95, 95% confidence interval CI 2.69–13.20, *p* < 0.001) and lower platelets level (risk ratio 0.992, 95% CI 0.986–0.998, *p* = 0.007) were independently associated with new CMBs.

**Table 6:** Multiple logistic regression analysis for new CMBs in the follow-up T2*-EPI.

	New CMBs in the follow up T2*-EPI		
**Risk factors**	**Adjusted risk ratio**	**95% CI**	***p*-value**
Presence of baseline CMBs	5.95	2.69–13.20	<0.001
Hypertension	5.45	0.99–29.90	0.051
Platelets	0.992	0.986–0.998	0.007
Observations	49

## Discussion

In our patient sample, administration of rtPA resulted in the appearance of new CMBs in nearly 30% of the subjects with AIS, and the baseline CMBs were associated with the higher risk of the new ones appearing after the treatment. However, in this cohort, the baseline CMB load was not related to the increased risk of hemorrhagic transformation, unlike in many of the previous reports (21, 23, 32, 39). This finding seems not to be in line with the pathophysiological connections of CMBs and various vascular pathologies, notably small vessel disease, which in turn is related to increased vascular vulnerability and thus with hemorrhagic transformation—these correlations have been extensively discussed elsewhere (21–23). The reason for this discrepancy might be that these associations may be influenced by many confounding factors connected with the individual characteristics of a patient. CMBs are to be found in “healthy” populations, however, the insight in the available reports prove susceptibility of these populations for numerous future health risks that are additionally modulated by many coexisting factors such as hypertension, smoking, Apo E homozygosity, aspirin intake, white matter lesions or cerebral amyloid angiopathy (40). In addition, it has been reported that the correlation between CMBs and hemorrhagic transformation is not linear and that there is a threshold of baseline CMBs that, only when exceeded, results in higher risks for the transformation, especially for the sICH. The most commonly reported threshold is the > 10 CMBs (41) and the definite majority of our patients presented with not more than 3 baseline CMBs.

One of the future health issues of patients with CMBs is cognitive decline. The available studies suggest strong correlations between CMBs and cognitive impairment (15, 16). The substantial correlation between rtPA treatment and new CMBs found in our study, may have a major implication for the cognitive health of patients with AIS, and thus, longitudinal long-term studies in rt-PA treated patients with AIS are warranted for the need of powerful assessment of potential relations between thrombolytic treatment, CMBs, and cognitive function. This seems to be specifically important in patients with minor and/or lacunar stroke, where thrombolytic treatment does not have that much scientific data, as large vessel occlusion strokes, to prove favorable outcomes (42).

The study was not controlled with a placebo sample and therefore the direct correlation of new CMBs with rtPA treatment cannot be concluded. Additionally, the acute cerebral infarction itself is suggested to promote the development of CMBs (43). However, taking into account other studies on CMBs in acute stroke, and the known rate of the hemorrhagic infarction unrelated to rtPA administration, it is unlikely that this burden value would be as high as about 30% with no connection to rtPA. Furthermore, a strong indication of rtPA-CMBs correlation has been presented in the recent study by Miwa et al., where new CMBs were found only in patients who received rtPA (31). The high percentage of new CMBs in our study is much higher in comparison with other studies that revealed rates in the range of 4–13% (27–31). We believe that it is probably connected with a small sample size that resulted in incidental recruitment of more predisposed patients, as we discussed earlier.

This study has several methodological limitations. Importantly, as discussed earlier, we do not have a control group of non-thrombolysis patients to compare with. Another limitation of this study was the use of a 1.5-T field MR machine which is inferior to a 3-T in detecting CMBs, and the T2^*^EPI-GRE imaging sequence—much shorter but less sensitive than SWI for CMBs detection (44, 45). To support the lower sensitivity of T2^*^EPI-GRE sequences, SWI sequences were additionally used to confirm the CMB assessments. Finally, our study encompassed a relatively small sample of subjects, which is, however, similar to many of the reported cohorts. The main reason for the small number of patients is the conflict of interest between the optimal timing of rtPA treatment and the demanding circumstances of MRI testing that is necessary for proper CMBs assessment.

In conclusion, in this study, we present that baseline CMBs burden in patients with AIS is associated with a higher risk of new CMBs after rtPA treatment, but not with the risk of hemorrhagic transformation. New CMBs alone or combined with other selected characteristics (e.g., age, platelet count, or creatine level as indicated in this study) may become a useful predictor of long-term CMBs-related complications of thrombolytic treatment of stroke.

## Data Availability Statement

The original contributions presented in the study are included in the article, further inquiries can be directed to the corresponding author.

## Ethics Statement

The studies involving human participants were reviewed and approved by the Independent Bioethics Committee for Scientific Research at Medical University of Gdańsk, Poland (Approval No. NKBBN/76/19). The participants provided their written informed consent to participate in this study. Written informed consent was obtained from the individuals for the publication of any potentially identifiable images or data included in this article.

## Author Contributions

BJ co-conceptualization of the study, patient recruitment, data assessment and analysis, combined analysis of all data, and writing. AG-G patient recruitment, data assessment and analysis, writing, and editing. DO neuroimaging data collection and assessment and writing. ES neuroimaging data assessment. AW statistical analysis and writing. BR clinical data assessment. BK conceptualization and funding receipt (a major project), writing, review, editing, and coordination. All authors contributed to the article and approved the submitted version.

## Funding

Neuroimaging data were part of our project funded by Siemens Heathineers (research grant for the Medical University of Gdansk, PI – BK, 2018; Contract Number C00229230). The authors declare that this study received funding from Siemens Heathineers. The funder was not involved in the study design, collection, analysis, interpretation of data, the writing of this article or the decision to submit it for publication.

## Conflict of Interest

The authors declare that the research was conducted in the absence of any commercial or financial relationships that could be construed as a potential conflict of interest.

## Publisher's Note

All claims expressed in this article are solely those of the authors and do not necessarily represent those of their affiliated organizations, or those of the publisher, the editors and the reviewers. Any product that may be evaluated in this article, or claim that may be made by its manufacturer, is not guaranteed or endorsed by the publisher.
